# Nurse participation in detecting signs of childhood autism in Primary Health Care

**DOI:** 10.1590/0034-7167-2023-0530

**Published:** 2025-03-10

**Authors:** Angelica Ribeiro Pinto de Oliveira, Liliane Faria da Silva, Tania Vignuda de Souza, Fernanda Garcia Bezerra Góes, Juliana Rezende Montenegro Medeiros de Moraes

**Affiliations:** I Universidade Federal do Rio de Janeiro. Rio de Janeiro, Rio de Janeiro, Brazil; II Universidade Federal Fluminense. Niterói, Rio de Janeiro, Brazil; III Universidade Federal Fluminense. Rio das Ostras, Rio de Janeiro, Brazil

**Keywords:** Child, Child Care, Autism Spectrum Disorder, Nursing, Office Nursing, Niño, Cuidado del Niño, Trastorno del Espectro Autista, Enfermería, Enfermería de Consulta

## Abstract

**Objectives:**

to understand nurse participation in the process of early detection of warning signs of autism spectrum disorders (ASD) in childcare consultations.

**Methods:**

qualitative, exploratory research, conducted through semi-structured interviews conducted between August and November 2022 with 27 nurses from family clinics in the city of Rio de Janeiro. The IRaMuTeQ® software was used for data treatment. Interpretations and theorizing were guided by Hildegard Peplau’s Theory of Interpersonal Relations.

**Results:**

lexical analysis pointed out thematic aspects related to the dynamics of development assessment, interpersonal relationship practices between nurses and family members as well as limits and interrelationships between healthcare professionals involved in early detection.

**Final Considerations:**

childcare consultations are characterized as a unique resource for the early detection of warning signs of ASD. Nurses need to be recognized as strategic agents in the face of this demand, especially in caring for socioeconomically vulnerable families.

## RESUMEN

**Objetivos:**

comprender la participación de los enfermeros en el proceso de detección temprana de signos de alerta de los trastornos del espectro autista (TEA) en las consultas de puericultura.

**Métodos:**

investigación cualitativa, exploratoria, realizada a través de entrevistas semiestructuradas realizadas entre agosto y noviembre de 2022 con 27 enfermeros de clínicas familiares de la ciudad de Río de Janeiro. Para procesar los datos se utilizó el *software* IRaMuTeQ®. Las interpretaciones y teorizaciones estuvieron guiadas por la Teoría de las Relaciones Interpersonales de Hildegard Peplau.

**Resultados:**

el análisis léxico destacó aspectos temáticos relacionados con la dinâmica de la evaluación del desarrollo, las prácticas de relación interpersonal entre enfermeros y familiares, así como los límites e interrelaciones entre los profesionales de salud involucrados en la detección temprana.

**Consideraciones Finales:**

las consultas de puericultura son un recurso único para la detección temprana de signos de alerta del TEA. Es necesario reconocer a las enfermeras como agentes estratégicos ante esta demanda, especialmente en el cuidado de famílias socioeconómicamente vulnerables.

## INTRODUCTION

Autism spectrum disorders (ASDs) are neurodevelopmental conditions that can be identified early in children under 36 months of age. The prevalence of ASDs is increasing worldwide, with significant implications for affected individuals and their families. ASDs are characterized by deficits in social communication and the presence of restricted or repetitive behaviors or interests. The etiology of ASDs is complex and not yet fully understood, involving genetic, neurobiological components and environmental exposures^(1)^.

In the United States of America, a recent study showed that there is one child with ASD for every 36 children^(2)^. Based on the projection of international data, and considering the Brazilian population of around 203 million inhabitants registered in the 2022 population census^(3)^, it is estimated that approximately 5.9 million people have ASD in the country^(4)^. It is worth noting that, in 2021, the Outpatient Information System (*Sistema de Informações Ambulatoriais*) identified 9.6 million visits to people with ASD in outpatient clinics, 4.1 million of which were related to children up to 9 years of age^(5)^.

The standard approach to diagnosing ASD involves early identification of warning signs during well-child visits by nurses. These signs, in children under 3 years of age, include little or no response to their own name when called, limited use of gestures in communication, difficulty developing imaginative play, little or no shared attention, difficulty maintaining eye contact, delayed speech, presence of repetitive and stereotyped movements, among other signs of developmental delay^(2, 6)^.

Although ASD diagnosis is clinical and performed by physicians, the multidisciplinary team’s work, especially in the identification of warning signs by nurses in childcare consultations in Primary Health Care (PHC), contributes significantly to early detection. This process should include direct observation of children’s behavior, interviews with family members and application of scales, questionnaires and standardized behavior assessment protocols^(6)^.

Research indicates that early detection of warning signs is essential for the diagnosis and appropriate treatment of ASD in childhood, significantly improving the prognosis and quality of life of children and their families. This tends to reduce waiting time for both diagnosis and the start of therapies, minimizing the emotional and financial impact on families and optimizing the service provided by health systems^(1, 5, 6)^.

In the context of PHC, nurses participate in detecting warning signs of ASD through monitoring child development, sensitively listening to family members’ reports and direct observation of children’s social interaction, communication and behavior. Moreover, nurses can establish effective interpersonal relationships to support and guide family members based on monitoring warning signs and identifying developmental delays, as well as how and when to seek support in case of suspicions, doubts or concerns^(7, 8)^. It is important to note that, depending on the child’s age, the identification of these signs during childcare consultations covers specific aspects related to social interaction, language, games and feeding^(5, 7, 8)^.

New research is needed to produce evidence that can demonstrate the strategic nurse participation in the early detection of warning signs of ASD, disseminate the use of standardized scales applicable by these professionals and develop plausible and timely interventions, especially for approaching children up to 36 months of age, whose brain plasticity can favor better prognoses and quality of life^(9, 10, 11, 12, 13)^.

Nurses, especially those working in the public health field, are often the first point of contact when families of a child with ASD seek diagnosis and support. Therefore, it is essential that these professionals know how to effectively identify the warning signs of ASD so that care is timely, sensitive, and evidence-based.

Considering the above, the following guiding question was raised: how does the process of early detection of warning signs of ASD occur in childcare consultations carried out by nurses, considering the perspective of interpersonal relationships?

To this end, Hildegard Elizabeth Peplau’s Theory of Interpersonal Relations was adopted as a reference, which considers the need to approach a positive therapeutic relationship between nurses and patients/family members during care experience. Peplau considers nursing an interpersonal, therapeutic, meaningful and educational process capable of establishing, through communication, the construction/evolution of an interactive dynamic with users, valuing their participation in their health situation and demanding self-knowledge from professionals^(14, 15)^.

## OBJECTIVES

To understand nurse participation in the process of early detection of warning signs of ASD in childcare consultations.

## METHODS

### Ethical aspects

The research was approved by the Research Ethics Committees of the proposing institution and the co-participating institution in April 2022. The Informed Consent Form was obtained from all participants. To maintain participant anonymity, alphanumeric codes were adopted (Nur 1, Nur 2, Nur 3, etc.).

### Study design

This is a qualitative and exploratory study, derived from an academic master’s dissertation developed at a federal university in the state of Rio de Janeiro, within the scope of the Children with Special Health Needs (CRIANES) research group, registered with the *Conselho Nacional de Desenvolvimento Científico e Tecnológico* (CNPq, Brazilian National Council for Scientific and Technological Development). To develop the study, the EQUATOR Network Consolidated criteria for REporting Qualitative research (COREQ) checklist was used.

### Study setting

The settings were five family clinics (FCs) in the city of Rio de Janeiro. The FCs adopt a model centered on Family Health Strategy, focused on prevention, health promotion and early diagnosis of diseases. They have multidisciplinary teams made up of physicians, nurses, nursing technicians, community health workers, health surveillance agents, dentists, oral health assistants and oral health technicians, offering outpatient care in various specialties. This setting established the requirements for data collection, including serving socioeconomically vulnerable families, most of whom do not have private health insurance and are therefore dependent on the *Sistema Único de Saúde* (SUS, Brazilian Health System), and who tend to possibly face greater challenges in the early detection of warning signs of ASD in their children.

To define the units, we sought to identify those that had the largest number of children registered for care. Based on this criterion, five clinics from programmatic areas (PAs) 4.0, 5.1 and 5.2 were selected, all located in the west zone of the city.

In the city of Rio de Janeiro, PAs are geographic and administrative divisions used to organize and manage healthcare services. Each PA is made up of a group of neighborhoods and health units, including hospitals, FCs, health centers, among others. This city is divided into ten PSs, numbered from PA 1.0 to PA 5.3. These areas are established based on epidemiological, demographic and socioeconomic criteria, aiming to optimize the distribution of resources and provision of healthcare services to the population. Each PA is responsible for planning, coordinating and executing public health actions, according to the specific needs of its region.

### Participant eligibility criteria

Twenty-seven nurses who provided childcare consultations at the aforementioned FCs participated in the study. These participants were recruited by unit managers, through on-site invitations and telephone contacts. Professionals whose practices were aimed at caring for children and providing childcare consultations and who had at least one year of experience in childcare were included. Nurses who were on vacation or on leave during the data collection phase were excluded. In total, 31 nurses were initially invited, however two refused to participate in the interviews, one was excluded for not meeting the eligibility criteria and one was on leave.

### Data collection and organization

Data collection was conducted by the main researcher, through semi-structured interviews, conducted in person, in rooms at FCs, from August to November 2022, without the presence of other people, without affecting the dynamics of services and outside participants’ working hours. The duration of interviews varied between 11 and 38 minutes (average of 19.9 minutes). Audio recording was made upon participants’ authorization in digital media format. To characterize the interviewees, a previously structured instrument was applied with closed-ended questions about demographic data, education and professional training, and open-ended questions about the phenomenon studied.

Data collection was concluded with theoretical saturation, defined based on repetition of information (manifest content) about the object of study and the exhaustion of the number of nurses who agreed to participate in the study in the selected FCs. There was homogeneity in the response pattern from interview number 21, with six more participants being included to confirm the absence of new data, which demonstrated sample saturation^(16, 17)^.

### Data analysis

After the interviews were completed, all the empirical material collected was transcribed and reviewed in order to obtain the documentary *corpus* for lexical analysis. The material was double-checked in the text *corpus* preparation, ensuring data anonymity, reliability and viability. Internal validity was ensured by data analysis by three CRIANES research group members, composed of researchers experienced in qualitative studies.

The textual corpus was processed using the *Interface de R pour les Analyses Multidimensionnelles de Textes et de Questionnaires* (IRaMuTeQ®), which allows different types of textual data analysis. For this research, we chose the Descending Hierarchical Classification (DHC), a Reinert method that classifies text segments according to their respective vocabularies, and the set of them is divided based on the frequency of reduced forms. DHC develops a category analysis by lexicon, through class structuring^(18)^.

The Theory of Interpersonal Relations was used both in the data collection instrument development and in data interpretation, providing a deeper understanding of the dynamics of interpersonal relationships in childcare consultations. In particular, the theory supported the approach to aspects of active participation, effective communication and meaningful interactions among nurses, family members and other healthcare professionals. These aspects were observed in guidance operationalization and identification phases of the therapeutic process, described by Hildegard Peplau, key moments for developing interventions aimed at the early detection of warning signs of ASD^(14)^.

## RESULTS

Of the 27 nurses participating in the research, 24 were female (88.9%) and three were male (11.1%), with ages ranging from 25 to 50 years (average of 36.3 years). All nurses had specialization and/or residency. The predominant area of training in specialization/residency was family health (22 nurses, 81.5%). It is noteworthy that the time since graduation in nursing among interviewees had an average of 9.5 years; the length working in the unit (FC) as a nurse had an average of 2.9 years; and the length working in child care had an average of 7.2 years.

**Figure 1:**
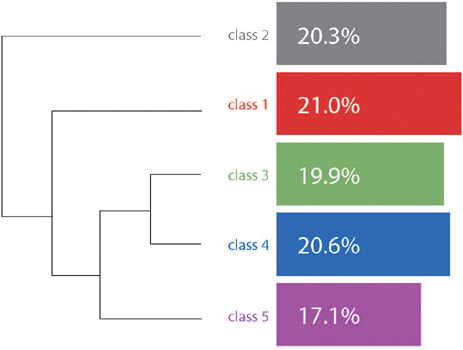
Descending Hierarchical Classification dendrogram

*Source: software IRaMuTeQ® 0.7 alpha 2.*

In total, IRaMuTeQº identified five classes through CHD, based on the organization of lexicons in the text *corpus* (Figure 1). The rate of use of interviews in the software demonstrated 1,086 classified segments out of a total of 1,395 (77.85%).

In this article, Class 2 is presented, which included 220 text segments (20.3%), which integrated a single thematic block, isolating itself from the other classes, as demonstrated in the following dendrogram, bringing together specific text segments that report a particular semantic pattern guided by the topic of this research. The most prevalent words in this class were “development”, “child’s card”, “assess”, “chart” and “milestone”. This class is the one that effectively answers the guiding question and objective outlined for this research. Its lexical elements encapsulate the nuances and particularities inherent to childcare consultations as an essential and strategic practice for the early detection of warning signs of ASD.

The following text segments express the dynamics of child-care consultations by nurses, demonstrating approaches to possible deviations in the development processes of children under their care:

*We record child growth and development assessment, which we mark using graphs at every consultation to see if it is within the correct reference for the age.* (Nur 8)

*We can’t make a diagnosis based on the child’s health record, but we can identify it. It has everything we need to assess the child, and everything is described there.* (Nur 10)

*And in the child’s card, I record the development indicators. Basically, the child’s card allows us to identify autism. It is a very helpful tool.* (Nur 14)

*So, if we don’t have this sensitivity, this different perspective, and don’t know exactly what the warning signs of autism are, we won’t be able to notice it, and this child will pass through and won’t reach the multidisciplinary team.* (Nur 12)

Below are text segments on interpersonal relationship practices between nurses and family members of children who attend childcare consultations at FCs:

*The interpersonal relationship with the families is very good. We create a bond. It’s not just a question of friendship, but it’s not just a technical issue either. We end up being the reference.* (Nur 17)

*We take care of the family. So, when the mother understands that the child really has delayed development, she comes to us with several questions.* (Nur 18)

*This ends up creating a relationship of trust, and it’s easier to approach them and tell them what we need to pay attention to, the warning signs of autism that need to be assessed. Then, I explain what the warning signs of autism are.* (Nur 24)

*Our relationship with the families of children who have the warning signs of autism is always about offering emotional support, support and guidance.* (Nur 14)

The following text segments demonstrate aspects of early detection of ASD warning signs, which involve nurse participation as well as interrelationships established with professionals from other areas.

*Cases of suspected autism warning signs never go directly to them* [multidisciplinary team members] *and then come to us. On the contrary, we nurses are the ones who first notice the problem, and we pass the case on to them.* (Nur 15)

*The role of a nurse in identifying autism warning signs is to communicate it to the team physician* (Nur 06).

*I can’t even make a diagnosis. I only identified the autism warning signs. I think that the difficulty we have today is this, of us arriving and having another assessment by a professional who can, together with us, make a diagnosis.* (Nur 10)

*When we call the physician in the office, and she notices something, she schedules this more thorough assessment, because they are better prepared to notice this, the speech therapist, the psychologist and everything else.* (Nur 12)

## DISCUSSION

During childcare consultations, a dialogic interaction between the nurse and the family is facilitated, including multidisciplinary team member participation. This favors the sharing of information, the construction of supportive relationships and the identification of possible delays in child development.

Nurse participation in childcare consultations observed in this study is in line with the scientific literature, which suggests that it should be centered on welcoming, active and qualified listening, open and frank dialogue, clear guidelines and the proposition of care^(9, 19)^. Undoubtedly, dialogical interaction between families and nurses is an important strategy for promoting child health, knowledge and assessment of growth and development, and disease prevention. This allows families to feel more cared for, welcomed and involved in the care process, especially when nuances that indicate the presence of disorders are observed. From the perspective of the Theory of Interpersonal Relations, dialogic interaction can be understood as a premise that determines a process in which the nurse and the family exchange information, experiences and knowledge, which allows the joint construction of solutions for children’s health problems^(14)^.

Some study participants highlighted the importance of a comprehensive child assessment during well-child visits through observation, interaction and assessment of physical, emotional and cognitive development. This unique opportunity, when carrying out well-child visits within the scope of PHC, allows for supporting actions for early detection of warning signs of ASD. As observed in this study and scientifically recommended, the information generated and collected during well-child visits should be recorded in the child’s health card, in the medical record and communicated to the family. These records provide a solid basis for monitoring child development over time, which makes it possible to identify important patterns and changes that indicate the need for early intervention. Added to this is the importance of operationalizing interpersonal relationships and facilitating effective communication between healthcare professionals, families and other child’s care team members, relevant to a collaborative and results-oriented approach^(20)^.

It is important to emphasize that nurses need to be able to intervene robustly and effectively in monitoring children, not just limiting themselves to measuring growth and development indicators. From this perspective of welcoming, the right to health and comprehensive care, these professionals must develop their consultations based on proactive interactions with children, their families and the communities where they live, understanding nursing as a dynamic profession based on helping people, especially those who are most vulnerable due to health inequities. Therefore, ongoing training is needed to develop a better standard of care for the population, which includes early detection and plausible interventions for children with suspected ASD and their families. Studies suggest that, in order to improve care for this type of demand, one way is to develop an educational process that focuses on this issue from the university training of healthcare professionals^(1, 6, 10, 21)^.

Although these are developmental disorders that begin in childhood, healthcare professionals working in PHC often point out difficulties in detecting early warning signs of ASD, especially nurses. Furthermore, it is known that early detection can guarantee a significant improvement in the levels of support for these children, which tends to reduce the stress of those involved in caring for them and the negative impacts that can interfere with family dynamics^(9)^.

Of the 27 nurses interviewed, none mentioned the use of scales with children under 36 months of age under their care to detect warning signs of ASD, as per guidelines from the Ministry of Health. The scientific literature describes the potential and advantages of using instruments and scales for this purpose. Among the validated instruments most widely used by nurses are M-CHAT, CARS, ESAT and DENVER II. Ideally, in addition to the instruments, the diagnostic process of ASD requires the employment of a multidisciplinary team with clinical experience, consisting of a nurse, pediatrician, child psychiatrist, psychologist, speech therapist and occupational therapist to ensure a more comprehensive view of the phenomenon under investigation^(22, 23, 24, 25)^.

Based on the reports of participants in this study, the process of detecting warning signs involves consultations and inter-consultations with different professionals, from which it can be inferred that nurses, since they are able to identify these signs during childcare consultations, have the responsibility of referring children for more in-depth assessment, when necessary. It is up to nurses to identify the warning signs of ASD early and physicians to make clinical diagnoses. It is also important to note that the care flows within the PHC setting play a fundamental role in the efficiency and effectiveness of the process of detecting the warning signs of ASD. As nurses work closely with other team members, there is a greater chance that referrals will be made in a timely manner so that children receive appropriate monitoring in the shortest possible time^(26)^.

Given the strategic role of nurses in the process of early detection of warning signs of ASDs within the scope of PHC, it is necessary to consider proposals that define specific competencies for the management of early detection together with professional oversight bodies for regulating such practice. Such an undertaking may establish greater legality and consequent development of training strategies more attentive to this purpose and, thus, provide greater support for this type of practice, clearer provision in the manuals and guidelines of the Ministry of Health, in addition to greater legitimacy to the roles that nurses need to play in view of the growing and alarming number of cases of ASD^(22)^.

In Brazil, studies point to difficulties related to the early detection of ASD cases in childhood. This is a population that is chronically unassisted by the health system, reflecting a disorderly expansion of diagnostics and resulting from the processes of medicalization of childhood. This creates tension in a struggle between the right to health and the construction of a network of expertise around ASD^(26, 27)^. This paradox lies in the need for the multidisciplinary team to develop skills and competencies that provide a basis for providing full care to children, especially those who are poorer and more vulnerable. To make matters worse, studies indicate that nurses do not feel fully prepared to care for children with ASD in clinical settings, including PHC, which involves these professionals not recognizing the possibilities for intervention^(28, 29)^.

An Indian study found that in developed countries, diagnosis is usually made by the age of 2, while in developing countries, the average age of children diagnosed is 4 to 5 years, with significant loss of time for early stimulation. Delayed diagnosis occurs due to insecurity among primary care professionals, late referrals, lack of knowledge of developmental milestones among nurses and physicians, inability of parents to raise critical developmental concerns, confusion of ASD with other conditions, and health systems that do not respond to the needs of underserved communities^(1)^.

In relation to the effective nurse participation, studies have revealed the importance of a call to action to promote innovative practices that provide for the incorporation of care technologies to facilitate reception actions, global assessment, screening and referral, health education and longitudinal monitoring of children with behavior patterns indicative of ASD^(30, 31)^. All of these aspects discussed here have the effect of demonstrating the need for investment in interventions that can, in fact, resolve these setbacks in the early detection of warning signs of ASD, especially within the scope of PHC.

### Study limitations

A limitation of this research was the development of interviews with nurses from the same Brazilian city (Rio de Janeiro), which may have generated homogeneity of data, due to the cultural and socioeconomic similarities in the work territories of these professionals, which tends to reduce the possibility of generalizations. This situation is related to the issue of data transferability, that is, the application of results to other contexts or groups that share similar characteristics to those studied, such as other regions of Brazil or countries with different health systems and distinct standards of care practices.

### Contributions to nursing, health or public policy

The strategic and unique relevance of well-child visits by nurses involves the implementation of measures that can be effective in the early detection of warning signs of ASD, with the potential to reduce cases of late diagnosis and its negative impacts. Furthermore, through positive interpersonal relationships with family members, the study also considers possible benefits to families, allowing them to seek help and services earlier, to ensure the well-being, health and quality of life of children.

For substantial advances in clinical practice and nursing research, measures must be taken to train professionals to recognize warning signs, strengthen communication skills and promote interpersonal relationships with families, structure specific and consistent protocols, strengthen teamwork and interdisciplinary collaboration for comprehensive and integrated support for families. Technological tools and software to support clinical decision-making should be incorporated, in addition to public policies that recognize and value the role of nurses in the early detection of warning signs of ASD, ensuring investments in adequate resources and infrastructure to support socioeconomically vulnerable families.

## FINAL CONSIDERATIONS

Nurse participation in the process of early detection of warning signs of ASD in childcare consultations, within the scope of PHC, is developed through interpersonal relationships with family members. In this regard, the development of childcare consultations provides nurses with a strategic approach that involves possibilities of identifying these warning signs, guiding referrals for consultations and interconsultations with multidisciplinary team members, with a view to structuring the diagnosis and defining early interventions.

The data highlighted the primary need to build and promote interpersonal relationships between nurses and families during childcare consultations, based on the development of help, trust, emotional support, monitoring, and the creation and strengthening of bonds.

It is necessary to structure proposals that strategically position nurses as one of the protagonists in the process of early detection of warning signs of ASD within the scope of PHC, in order to confer legality and legitimacy to their practice. This can favor the engagement of these professionals in more sustainable health policies and practices, ensuring a better quality of life for children with special health needs and their families, especially in caring for the most socioeconomically vulnerable.
