# Supplementary material for: Nurse participation in detecting signs of childhood autism in Primary Health Care
Source: Rev Bras Enferm. 2025 Mar 10;78(1):e20230530. doi: 10.1590/0034-7167-2023-0530 (PMC11893064; doi:10.1590/0034-7167-2023-0530)
Supplement: Translation

# Translation: Participação de enfermeiros na detecção de sinais de autismo infantil na Atenção Primária à Saúde

**DOI:** 10.1590/0034-7167-2023-0530pt

**Authors:** Angelica Ribeiro Pinto de Oliveira, Liliane Faria da Silva, Tania Vignuda de Souza, Fernanda Garcia Bezerra Góes, Juliana Rezende Montenegro Medeiros de Moraes

**Affiliations:** I Universidade Federal do Rio de Janeiro. Rio de Janeiro, Rio de Janeiro, Brasil; II Universidade Federal Fluminense. Niterói, Rio de Janeiro, Brasil; I Universidade Federal Fluminense. Rio das Ostras, Rio de Janeiro, Brasil

**Keywords:** Criança, Cuidado da Criança, Transtorno do Espectro Autista, Enfermagem, Enfermagem no Consultório

## Abstract

**Objetivos:**

compreender a participação de enfermeiros no processo de detecção precoce dos sinais de alerta dos transtornos do espectro autista (TEA) em consultas de puericultura.

**Métodos:**

pesquisa qualitativa, exploratória, conduzida por entrevistas semiestruturadas realizadas entre agosto e novembro de 2022 com 27 enfermeiros de clínicas da família do município do Rio de Janeiro. Utilizou-se o *software* IRaMuTeQ® para tratamento dos dados. As interpretações e teorização foram guiadas pela Teoria das Relações Interpessoais de Hildegard Peplau.

**Resultados:**

a análise lexical apontou aspectos temáticos relacionados à dinâmica da avaliação do desenvolvimento, práticas de relacionamento interpessoal entre enfermeiros e familiares, além de limites e interrelações entre os profissionais de saúde envolvidos na detecção precoce.

**Considerações Finais:**

as consultas de puericultura caracterizam-se como recurso singular para a detecção precoce dos sinais de alerta dos TEA. Enfermeiros precisam ser reconhecidos como agentes estratégicos diante dessa demanda, especialmente no cuidado às famílias vulnerabilizadas socioeconomicamente.

## INTRODUÇÃO

Os transtornos do espectro autista (TEA) são condições do neurodesenvolvimento que podem ser identificadas precocemente em crianças com idade inferior a 36 meses. A prevalência dos TEA está crescendo em todo o mundo, apresentando implicações consideráveis para os indivíduos afetados e suas famílias. Os TEA são caracterizados por déficits na comunicação social e pela presença de comportamentos ou interesses restritos ou repetitivos. A etiologia dos TEA é complexa e ainda não completamente compreendida, envolvendo componentes genéticos, neurobiológicos e exposições ambientais^(1)^.

Nos Estados Unidos da América, estudo recente apontou a proporção de uma criança com TEA para cada 36 crianças^(2)^. Com base na projeção de dados internacionais, e considerando a população brasileira de cerca de 203 milhões de habitantes registrada no censo populacional de 2022^(3)^, estima-se que aproximadamente 5,9 milhões de pessoas tenham TEA no país^(4)^. Cabe ressaltar que, em 2021, o Sistema de Informações Ambulatoriais identificou 9,6 milhões de atendimentos a pessoas com TEA em ambulatórios, sendo 4,1 milhões relacionados ao público infantil com até 9 anos de idade^(5)^.

A abordagem padrão para o diagnóstico de TEA consiste na identificação precoce de sinais de alerta durante as consultas de puericultura realizadas pelos enfermeiros. Esses sinais, em crianças menores de 3 anos, incluem pouca ou nenhuma resposta ao próprio nome quando chamadas, uso limitado de gestos na comunicação, dificuldade de desenvolver brincadeiras imaginativas, baixa ou nenhuma atenção compartilhada, dificuldade de manter o olhar, atraso na fala, presença de movimento repetitivos e estereotipados, entre outros sinais de atraso no desenvolvimento^(2, 6)^.

Embora o diagnóstico dos TEA seja clínico e realizado por médicos, o trabalho da equipe multidisciplinar, especialmente na identificação dos sinais de alerta pelos enfermeiros nas consultas de puericultura na Atenção Primária à Saúde (APS), contribui significativamente para a detecção precoce. Esse processo deve englobar a observação direta do comportamento da criança, entrevistas com familiares e a aplicação de escalas, questionários e protocolos padronizados de avaliação do comportamento^(6)^.

Pesquisas indicam que a detecção precoce dos sinais de alerta é fundamental para o diagnóstico e tratamento adequado dos TEAs na infância, melhorando significativamente o prognóstico e a qualidade de vida das crianças e suas famílias. Isso tende a reduzir o tempo de espera tanto do diagnóstico quanto do início das terapias, minimizando o impacto emocional e financeiro sobre as famílias e otimizando o serviço prestado pelos sistemas de saúde^(1, 5, 6)^.

No contexto da APS, a participação dos enfermeiros na detecção dos sinais de alerta dos TEA ocorre através do acompanhamento do desenvolvimento infantil, da escuta sensível dos relatos dos familiares e da observação direta da interação social, comunicação e comportamento da criança. Além disso, os enfermeiros podem estabelecer relações interpessoais efetivas para suporte e orientação aos familiares baseadas no acompanhamento dos sinais de alerta e identificação de atrasos no desenvolvimento, bem como sobre como e quando buscar apoio em caso de suspeitas, dúvidas ou preocupações^(7, 8)^. Destaca-se que, em função da idade da criança, a identificação desses sinais durante as consultas de puericultura abrange aspectos específicos relativos à interação social, linguagem, brincadeiras e alimentação^(5, 7, 8)^.

Novas pesquisas são necessárias para produzir evidências que possam demonstrar a participação estratégica dos enfermeiros na detecção precoce dos sinais de alerta dos TEAs, difundir o uso de escalas padronizadas aplicáveis por esses profissionais e desenvolver intervenções plausíveis e oportunas, especialmente para abordagem de crianças com até 36 meses de idade, cuja plasticidade cerebral pode favorecer melhores prognósticos e qualidade de vida^(9, 10, 11, 12, 13)^.

Os enfermeiros, especialmente aqueles que trabalham no campo da saúde pública, são frequentemente o primeiro ponto de contato quando os familiares de uma criança com TEA procuram diagnóstico e apoio. Portanto, é essencial que esses profissionais saibam identificar efetivamente os sinais de alerta dos TEAs, para que os cuidados sejam oportunos, sensíveis e baseados em evidências.

Diante do exposto, foi traçada a seguinte questão norteadora: como se dá o processo de detecção precoce dos sinais de alerta dos TEAs nas consultas de puericultura realizadas pelos enfermeiros, considerando a perspectiva das relações interpessoais?

Para tanto, adotou-se como referencial a Teoria da Relações Interpessoais de Hildegard Elizabeth Peplau, que considera a necessidade de abordagem de uma relação terapêutica positiva entre enfermeiros e pacientes/familiares durante a experiência do cuidado. Peplau considera a enfermagem um processo interpessoal, terapêutico, significativo e educativo capaz de estabelecer, por meio da comunicação, a construção/evolução de uma dinâmica interativa com o usuário, valorizando sua participação na sua situação de saúde e demandando autoconhecimento do profissional^(14, 15)^.

## OBJETIVOS

Compreender a participação de enfermeiros no processo de detecção precoce dos sinais de alerta dos TEAs em consultas de puericultura.

## MÉTODOS

### Aspectos éticos

A pesquisa foi aprovada pelos Comitês de Ética em Pesquisa da instituição proponente e da instituição coparticipante em abril de 2022. O Termo de Consentimento Livre e Esclarecido foi obtido de todos os participantes. Para manter o anonimato dos mesmos, foram adotados códigos alfanuméricos (Enf 1, Enf 2, Enf 3, etc.).

### Desenho do estudo

Trata-se de estudo de abordagem qualitativa, de tipologia exploratória, derivado de uma dissertação de mestrado acadêmico desenvolvida em uma universidade federal do estado do Rio de Janeiro, no âmbito do grupo de pesquisa Crianças com Necessidades de Saúde Especiais (CRIANES), cadastrado no Conselho Nacional de Desenvolvimento Científico e Tecnológico (CNPq). Para o desenvolvimento do estudo, utilizou-se o *checklist Consolidated criteria for Reporting Qualitative research* (COREQ) da Rede EQUATOR.

### Cenários do estudo

Os cenários foram cinco clínicas da família (CFs) do município do Rio de Janeiro. As CFs adotam um modelo centrado na Estratégia Saúde da Família, focado em ações de prevenção, promoção da saúde e diagnóstico precoce de doenças. Possuem equipes multidisciplinares compostas por médicos, enfermeiros, técnicos de enfermagem, agentes comunitários de saúde, agentes de vigilância em saúde, dentistas, auxiliares de saúde bucal e técnicos de saúde bucal, oferecendo atendimento ambulatorial em diversas especialidades. Esse cenário estabeleceu os requisitos para a coleta de dados, inclusive por atender famílias socioeconomicamente vulneráveis, a maioria sem planos de saúde privado e, portanto, dependentes do Sistema Único de Saúde (SUS), e que tendem a possivelmente enfrentar maiores desafios na detecção precoce dos sinais de alerta dos TEAs de suas crianças.

Para a definição das unidades, buscou-se identificar aquelas que possuíam o maior número de crianças cadastradas para atendimento. Com base nesse critério, foram selecionadas cinco clínicas das áreas programáticas (APs) 4.0, 5.1 e 5.2, todas localizadas na zona oeste da cidade.

No município do Rio de Janeiro, as APs são divisões geográficas e administrativas utilizadas para a organização e gestão dos serviços de saúde. Cada AP é composta por um conjunto de bairros e unidades de saúde, incluindo hospitais, CFs, centros de saúde, entre outros. Esse município é dividido em dez APs, numeradas de AP 1.0 a AP 5.3. Essas áreas são estabelecidas com base em critérios epidemiológicos, demográficos e socioeconômicos, visando otimizar a distribuição dos recursos e a prestação de serviços de saúde à população. Cada AP tem a responsabilidade de planejar, coordenar e executar ações de saúde pública, conforme as necessidades específicas de sua região.

### Critérios de elegibilidade dos participantes

Participaram do estudo 27 enfermeiros que realizavam consultas de puericultura nas CFs mencionadas. A captação desses participantes foi realizada por indicação dos gestores das unidades, através de convites *in loco* e contatos telefônicos. Foram incluídos profissionais cujas práticas fossem dirigidas ao atendimento de crianças e realização de consultas de puericultura e que possuíssem experiência de pelo menos um ano na assistência à infância. Foram excluídos enfermeiros em período de férias ou em licença durante a fase de coleta de dados. No total, 31 enfermeiros foram inicialmente convidados, entretanto dois se recusaram a participar das entrevistas, uma foi excluída por não atender aos critérios de elegibilidade e uma estava em período de licença.

### Coleta e organização dos dados

A coleta de dados foi conduzida pela pesquisadora principal, por meio de entrevistas semiestruturadas, realizadas de forma presencial, em salas nas CFs, no período de agosto a novembro de 2022, sem a presença de outras pessoas, sem prejuízo à dinâmica dos atendimentos e fora do horário de trabalho dos participantes. A duração das entrevistas variou entre 11 e 38 minutos (média de 19,9 minutos). A gravação de áudio foi realizada após autorização dos participantes em formato de mídia digital. Para a caracterização dos entrevistados, foi aplicado um instrumento previamente estruturado com perguntas fechadas sobre dados demográficos, de formação e de capacitação profissional, e perguntas abertas sobre o fenômeno estudado.

A coleta de dados foi encerrada com a saturação teórica, definida a partir da repetição de informações (conteúdo manifesto) sobre o objeto do estudo e pelo esgotamento do número de enfermeiros que aceitaram participar do estudo nas CFs selecionadas. Houve homogeneidade do padrão de resposta a partir da entrevista de número 21, sendo incluídos mais seis participantes para confirmar a ausência de novos dados, o que demonstrou a saturação amostral^(16, 17)^.

### Análise dos dados

Após o término das entrevistas, foram realizadas transcrições e revisões de todo o material empírico coletado, a fim de se obter o *corpus* documental para análise lexical. O material passou por dupla checagem na preparação do *corpus* textual, garantindo o anonimato, a confiabilidade e a viabilidade dos dados. A validade interna foi assegurada pela análise dos dados por três membros do grupo de pesquisa CRIANES, composto por pesquisadores experientes em estudos de abordagem qualitativa.

O *corpus* textual foi tratado por meio do *software Interface de R pour les Analyses Multidimensionnelles de Textes et de Questionnaires* (IRaMuTeQ®), que permite distintos tipos de análise de dados textuais. Para esta pesquisa, optou-se pela Classificação Hierárquica Descendente (CHD), método de Reinert que classifica os segmentos de texto em função dos seus respectivos vocabulários, sendo o conjunto deles repartido com base na frequência das formas reduzidas. A CHD desenvolve uma análise de categorias por léxico, através da estruturação de classes^(18)^.

A Teoria de Relações Interpessoais foi empregada tanto na elaboração do instrumento de coleta quanto na interpretação dos dados, proporcionando uma compreensão mais profunda da dinâmica das relações interpessoais nas consultas de puericultura. Em particular, a teoria apoiou a abordagem de aspectos sobre participação ativa, comunicação efetiva e interações significativas entre enfermeiros, familiares e outros profissionais de saúde. Esses aspectos foram observados na operacionalização das fases de orientação e identificação do processo terapêutico, descritas por Hildegard Peplau, momentos-chave para o desenvolvimento de intervenções voltadas à deteção precoce dos sinais de alerta dos TEAs^(14)^.

## RESULTADOS

Dos 27 enfermeiros participantes da pesquisa, 24 foram do sexo feminino (88,9%) e três do sexo masculino (11,1%), com idades variando entre 25 e 50 anos (média de 36,3 anos). Todos os enfermeiros possuíam especialização e/ou residência. A área predominante de formação em especialização/residência foi a de saúde da família (22 enfermeiros, 81,5%). Destaca-se que o tempo de formação de graduação em enfermagem entre os entrevistados obteve média de 9,5 anos; o tempo de trabalho na unidade (CF) como enfermeiro obteve média de 2,9 anos; e o tempo de trabalho na assistência a crianças obteve média de 7,2 anos.

Ao todo, o IRaMuTeQ® identificou cinco classes por meio da CHD, em função da organização dos léxicos do *corpus* textual (Figura 1). A taxa de aproveitamento das entrevistas no *software* demonstrou 1.086 segmentos classificados de um total de 1.395 (77,85 %).

**Figura 1:** Dendrograma da Classificação Hierárquica Descendente

*Fonte: software IRaMuTeQ® 0.7 alpha 2.*

Neste artigo, está apresentada a Classe 2, que contemplou 220 segmentos de texto (20,3%), a qual integrou um único bloco temático, isolando-se das demais classes, conforme demonstrado no dendrograma a seguir, reunindo segmentos de texto específicos que reportam um padrão semântico particular orientado pelo tema desta pesquisa. As palavras com maior prevalência nesta classe foram “desenvolvimento”, “caderneta da criança”, “avaliar”, “gráfico” e “marco”. Essa classe é a que responde efetivamente à questão norteadora e objetivo delineados para esta pesquisa. Seus elementos lexicais encapsulam as nuances e particularidades inerentes às consultas de puericultura enquanto prática essencial e estratégica para a detecção precoce dos sinais de alerta dos TEAs.

Os segmentos de texto a seguir expressam a dinâmica das consultas de puericultura pelos enfermeiros, demonstrando enfoques acerca de possíveis desvios dos processos de desenvolvimento das crianças sob os seus cuidados:

*Registramos a avaliação do crescimento e desenvolvimento da criança, o que a gente marca através dos gráficos em toda consulta para ver se está dentro da referência certa para a idade.* (Enf 8)

*A gente não tem como fechar um diagnóstico pela caderneta da criança, mas identificar, a gente tem como identificar. Nela tem tudo o que a gente precisa para avaliação da criança, e vem tudo descrito ali.* (Enf 10)

*E na caderneta da criança, vou registrando os indicadores de desenvolvimento. Basicamente, a caderneta da criança nos permite identificar o autismo. Ela é um instrumento que ajuda bastante.* (Enf 14)

*Então, se a gente não tiver essa sensibilidade, esse olhar diferenciado, e não souber direito quais são os sinais de alerta do autismo, não dá para perceber, e essa criança vai passar, e não vai chegar na equipe multiprofissional.* (Enf 12)

Na sequência, estão apresentados segmentos de texto sobre práticas de relacionamento interpessoal entre enfermeiros e membros da família das crianças que frequentam as consultas de puericultura nas CFs:

*O relacionamento interpessoal com as famílias é muito bom. A gente cria vínculo. Não chega a ser uma questão de amizade, mas também não é uma questão técnica somente. A gente acaba sendo a referência.* (Enf 17)

*Nós vamos cuidando da família. Então, quando a mãe entende que realmente a criança tem um desenvolvimento atrasado, ela já chega para nós com diversas perguntas.* (Enf 18)

*Acaba criando uma relação de confiança, e fica mais fácil chegar e dizer o que precisamos atentar, os sinais de alerta do autismo que precisam ser avaliados. Aí, eu explico o que são os sinais de alerta do autismo.* (Enf 24)

*O nosso relacionamento com a família das crianças, que possuem os sinais de alerta do autismo, é sempre no sentido de oferecer suporte emocional, apoio e orientações.* (Enf 14)

Os próximos segmentos de texto demonstram aspectos da detecção precoce dos sinais de alerta dos TEAs, que envolvem a participação do enfermeiro, bem como as interrelações estabelecidas junto a profissionais de outras áreas.

*Os casos de suspeita de sinais de alerta do autismo nunca passam direto para eles* [membros da equipe multiprofissional], *e depois vêm para a gente. Ao contrário, nós enfermeiros é que percebemos primeiro o problema e passamos o caso para eles.* (Enf 15)

*O papel como enfermeira na identificação dos sinais de alerta do autismo é de comunicar ao médico da equipe.* (Enf 06)

*Eu não posso nem fechar diagnóstico. Eu somente identifiquei os sinais de alerta do autismo. Eu acho que a dificuldade que a gente tem hoje é isso, de a gente chegar e ter uma outra avaliação de um profissional que possa ali, junto com a gente, fechar um diagnóstico.* (Enf 10)

*Quando a gente chama a médica no consultório, e ela percebe alguma coisa, ela já agenda essa avaliação mais criteriosa, porque eles têm um preparo maior para perceber isso, a fonoaudióloga, a psicóloga e tudo mais.* (Enf 12)

## DISCUSSÃO

Durante as consultas de puericultura, viabiliza-se a construção de uma interação dialógica entre o enfermeiro e a família, incluindo a participação de membros da equipe multiprofissional. Isso favorece o compartilhamento de informações, a construção de relações de ajuda e a identificação de possíveis atrasos no desenvolvimento da criança.

A participação do enfermeiro nas consultas de puericultura observada neste estudo está em consonância com a literatura cientifica, que sugere que deve estar centrada no acolhimento, na escuta ativa e qualificada, no diálogo aberto e franco, em orientações claras e na proposição de cuidados^(9, 19)^. Indubitavelmente, a interação dialógica entre famílias e enfermeiros é uma estratégia importante para a promoção da saúde infantil, conhecimento e avaliação do crescimento e desenvolvimento e prevenção de doenças. Isso permite que as famílias se sintam mais cuidadas, acolhidas e envolvidas no processo assistencial, especialmente quando são observadas nuances que indiquem a presença de transtornos. Na perspectiva da Teoria das Relações Interpessoais, a interação dialógica pode ser compreendida como premissa que determina um processo em que o enfermeiro e a família trocam informações, experiências e conhecimentos, o que permite a construção conjunta de soluções para os problemas de saúde da criança^(14)^.

Alguns participantes do estudo apontaram a importância da avaliação global da criança durante as consultas de puericultura através da observação, interação e avaliação do desenvolvimento físico, emocional e cognitivo. Essa oportunidade singular, na realização das consultas de puericultura no âmbito da APS, permite respaldar ações de detecção precoce dos sinais de alerta dos TEAs. Assim como observado neste estudo e preconizado cientificamente, as informações geradas e coletadas nas consultas de puericultura devem ser registradas na caderneta de saúde da criança, no prontuário e comunicadas à família. Esses registros fornecem uma base sólida para o acompanhamento do desenvolvimento da criança ao longo do tempo, o que possibilita a identificação de padrões e mudanças importantes que indicam a necessidade de intervenção precoce. Somam-se a isso a importância da operacionalização de relações interpessoais e a facilitação da comunicação eficaz entre os profissionais de saúde, famílias e outros membros da equipe de cuidados da criança, relevantes para uma abordagem colaborativa e orientada para resultados^(20)^.

É necessário ressaltar que os enfermeiros precisam ser capazes de intervir de forma robusta e efetiva no acompanhamento das crianças, não se restringindo apenas à mensuração de indicadores de crescimento e desenvolvimento. Nessa perspectiva de acolhimento, direito à saúde e integralidade do cuidado, esses profissionais devem desenvolver suas consultas a partir de interações proativas com a criança, sua família e a comunidade onde vivem, na compreensão da enfermagem como uma profissão dinâmica e fundamentada na relação de ajuda às pessoas, especialmente aquelas mais vulneráveis, devido às iniquidades em saúde. Portanto, é preciso capacitação constante para o desenvolvimento de um melhor padrão de atendimento à população, o que inclui a detecção precoce e intervenções plausíveis junto às crianças com suspeita de TEA e às suas famílias. Estudos propõem que, para qualificar o atendimento a esse tipo de demanda, uma das formas é o desenvolvimento de um processo educativo que enfoque tal problemática desde a formação universitária dos profissionais de saúde^(1, 6, 10, 21)^.

Embora sejam transtornos de desenvolvimento, que se iniciam na infância, comumente os profissionais de saúde que atuam na APS apontam dificuldades na detecção precoce dos sinais de alerta dos TEAs, em especial os enfermeiros. Ademais, sabe-se que a detecção precoce pode vir a garantir uma melhora significativa dos níveis de suporte dessas crianças, o que tende a reduzir o estresse das pessoas envolvidas no cuidado e os impactos negativos que podem intervir na dinâmica familiar^(9)^.

Dos 27 enfermeiros entrevistados, nenhum mencionou a aplicação de escalas junto às crianças menores de 36 meses de idade sob os seus cuidados para a detecção dos sinais de alerta dos TEAs, conforme orientações do Ministério da Saúde. A literatura científica descreve as potencialidades e vantagens do uso de instrumentos e escalas para esse propósito. Entre os instrumentos validados e de maior aplicação por enfermeiros, estão o M-CHAT, a escala CARS, o questionário ESAT e o DENVER II. Idealmente, além dos instrumentos, o processo diagnóstico dos TEA requer a empregabilidade de uma equipe multidisciplinar, com experiência clínica, composta por enfermeiro, pediatra, psiquiatra infantil, psicólogo, fonoaudiólogo e terapeuta ocupacional, para garantir uma visão mais abrangente sobre o fenômeno investigado^(22, 23, 24, 25)^.

A partir dos relatos dos participantes deste estudo, o processo de detecção dos sinais de alerta envolve consultas e interconsultas com diferentes profissionais, do que se depreende que os enfermeiros, por estarem em condições para identificar esses sinais já durante as consultas de puericultura, têm a responsabilidade de encaminhar as crianças para avaliação mais aprofundada, quando for necessário. Cabe ao enfermeiro a identificação precoce dos sinais de alerta dos TEAs, e ao médico, o diagnóstico clínico. Ressalta-se ainda que os fluxos de atendimento no âmbito da APS têm papel fundamental na eficiência e eficácia do processo de detecção dos sinais de alerta dos TEAs. Na medida em que os enfermeiros trabalham em estreita colaboração com outros membros da equipe, há maiores chances de que os encaminhamentos sejam feitos de forma oportuna para que as crianças recebam o acompanhamento adequado no menor tempo possível^(26)^.

Em função do papel estratégico de enfermeiros no processo de detecção precoce dos sinais de alerta dos TEAs no âmbito da APS, há que se pensar em proposições que definam competências específicas para o manejo da detecção precoce junto aos órgãos de fiscalização profissional para a regulamentação de tal prática. Tal empreendimento pode vir a estabelecer maior legalidade e consequente desenvolvimento de estratégias de formação mais atentas a esse propósito e, assim, dar maior sustentação a esse tipo de prática, previsão mais clara nos manuais e orientações do Ministério da Saúde, além de maior legitimidade aos papéis que o enfermeiro precisa desempenhar diante do número crescente e alarmante de casos de TEA^(22)^.

No Brasil, estudos apontam dificuldades relacionadas à detecção precoce de casos de TEA na infância. Essa é uma população cronicamente desassistida pelo sistema de saúde, reflexo de uma expansão diagnóstica desordenada e resultante dos processos de medicalização da infância. Isso tensiona uma luta entre o direito à saúde e a construção de uma rede de *expertise* em torno dos TEAs^(26, 27)^. Nesse paradoxo, reside a necessidade de construção de habilidades e competências pela equipe multiprofissional que deem base de sustentação para o cuidado pleno às crianças, especialmente àquelas mais empobrecidas e vulneráveis. Como agravante, estudos apontam que os enfermeiros não se sentem plenamente preparados para cuidar de crianças com TEA em ambientes clínicos, incluindo a APS, o que envolve o não reconhecimento de possibilidades de intervenção por parte desses profissionais^(28, 29)^.

Estudo indiano identificou que, nos países desenvolvidos, o diagnóstico geralmente é realizado até os 2 anos de idade, enquanto que, nos países em desenvolvimento, a média de idade das crianças diagnosticadas é de 4 a 5 anos, com perda importante de tempo de estimulação precoce. O atraso no diagnóstico ocorre devido à insegurança dos profissionais da atenção primária, encaminhamentos tardios, falta de conhecimento de enfermeiros e médicos sobre os marcos do desenvolvimento, incapacidade dos pais de levantar preocupações críticas de desenvolvimento, confusão dos TEAs com outras condições e sistemas de saúde que não respondem às necessidades das comunidades carentes^(1)^.

Sobre a participação eficaz de enfermeiros, estudos vêm revelando a importância de um chamado à ação para a promoção de práticas inovadoras que prevejam a incorporação de tecnologias de cuidado para facilitar ações de acolhimento, avaliação global, triagem e encaminhamento, educação em saúde e acompanhamento longitudinal das crianças com padrões de comportamento indicativos de TEA^(30, 31)^. Todos esses aspectos ora abordados têm o efeito de demonstrar a necessidade de investimento em intervenções que, de fato, possam resolver esses percalços na detecção precoce dos sinais de alerta dos TEAs, especialmente no âmbito das APS.

### Limitações do estudo

Uma limitação desta pesquisa foi o desenvolvimento de entrevistas com enfermeiros de um mesmo município brasileiro (Rio de Janeiro), o que pode ter gerado homogeneidade dos dados, devido às similaridades culturais e socioeconômicas nos territórios de trabalho desses profissionais, o que tende a minorar a possibilidade de generalizações. Tal situação guarda relação com a questão da transferibilidade dos dados, ou seja, com a aplicação dos resultados a outros contextos ou grupos que compartilhem características semelhantes às estudadas, como outras regiões do Brasil ou países com diferentes sistemas de saúde e padrões distintos de práticas assistenciais.

### Contribuições para as áreas da enfermagem, saúde ou políticas públicas

A relevância estratégica e singular das consultas de puericultura por enfermeiros envolve a implementação de medidas que podem ser efetivas na detecção precoce dos sinais de alerta dos TEAs, com potencial para reduzir os casos de diagnósticos tardios e seus impactos negativos. Além disso, por meio de relações interpessoais positivas com os familiares, o estudo também considera possíveis benefícios às famílias, permitindo-lhes buscar ajuda e serviços mais cedo, para garantir o bem-estar, a saúde e a qualidade de vida das crianças.

Para avanços substanciais na prática clínica e na pesquisa em enfermagem, deve-se empreender medidas de capacitação dos profissionais para o reconhecimento dos sinais de alerta, fortalecimento das habilidades de comunicação e promoção do relacionamento interpessoal com as famílias, estruturação de protocolos específicos e consistentes, fortalecimento do trabalho em equipe e colaboração interdisciplinar para um suporte abrangente e integrado às famílias. Devem ser incorporadas ferramentas tecnológicas e *softwares* de apoio à decisão clínica, além de políticas públicas que reconheçam e valorizem o papel dos enfermeiros na detecção precoce dos sinais de alerta dos TEAs, garantindo investimentos em recursos e infraestrutura adequados para o suporte às famílias vulneráveis socioeconomicamente.

## CONSIDERAÇÕES FINAIS

A participação de enfermeiros no processo de detecção precoce dos sinais de alerta dos transtornos dos TEAs em consultas de puericultura, no âmbito da APS, desenvolve-se por meio de relações interpessoais com os familiares. Nesse sentido, o desenvolvimento das consultas de puericultura proporciona aos enfermeiros uma abordagem estratégica que envolve possibilidades de identificação desses sinais de alerta, orientando encaminhamentos para consultas e interconsultas com membros da equipe multidisciplinar, com vistas a ser estruturado o diagnóstico e serem definidas intervenções precoces.

Os dados apontaram a necessidade primaz de construção e promoção de relações interpessoais entre os enfermeiros e as famílias durante as consultas de puericultura, com base no desenvolvimento da ajuda, confiança, suporte emocional, acompanhamento, criação e fortalecimento de vínculos.

É necessário estruturar proposições que posicionem estrategicamente o enfermeiro como um dos protagonistas no processo de detecção precoce dos sinais de alerta dos TEA no âmbito da APS, a fim de conferir legalidade e legitimidade à sua prática. Isso pode favorecer o engajamento desses profissionais em políticas e práticas de saúde mais sustentáveis, garantindo maior qualidade de vida para as crianças com necessidade de saúde especiais e suas famílias, especialmente no cuidado às mais vulneráveis socioeconomicamente.
